# The role of cerebral blood flow gradient in peritumoral edema for differentiation of glioblastomas from solitary metastatic lesions

**DOI:** 10.18632/oncotarget.12053

**Published:** 2016-09-15

**Authors:** Lin Lin, Yunjing Xue, Qing Duan, Bin Sun, Hailong Lin, Xinming Huang, Xiaodan Chen

**Affiliations:** ^1^ Department of Radiology, Union Hospital of Fujian Medical University, Fuzhou, Fujian, China; ^2^ Department of Radiology, Fujian Provincial Cancer Hospital, Fuzhou, Fujian, China

**Keywords:** three-dimensional arterial spin labeling, cerebral blood flow, perfusion, glioblastoma, metastasis

## Abstract

**Objective:**

Differentiation of glioblastomas from solitary brain metastases using conventional MRI remains an important unsolved problem. In this study, we introduced the conception of the cerebral blood flow (CBF) gradient in peritumoral edema—the difference in CBF values from the proximity of the enhancing tumor to the normal-appearing white matter, and investigated the contribution of perfusion metrics on the discrimination of glioblastoma from a metastatic lesion.

**Materials and Methods:**

Fifty-two consecutive patients with glioblastoma or a solitary metastatic lesion underwent three-dimensional arterial spin labeling (3D-ASL) before surgical resection. The CBF values were measured in the peritumoral edema (near: G1; Intermediate: G2; Far: G3). The CBF gradient was calculated as the subtractions CBF_G1_ –CBF_G3_, CBF_G1_ – CBF_G2_ and CBF_G2_ – CBF_G3_. A receiver operating characteristic (ROC) curve analysis was used to seek for the best cutoff value permitting discrimination between these two tumors.

**Results:**

The absolute/related CBF values and the CBF gradient in the peritumoral regions of glioblastomas were significantly higher than those in metastases(*P* < 0.038). ROC curve analysis reveals, a cutoff value of 1.92 ml/100g for the CBF gradient of CBF_G1_ –CBF_G3_ generated the best combination of sensitivity (92.86%) and specificity (100.00%) for distinguishing between a glioblastoma and metastasis.

**Conclusion:**

The CBF gradient in peritumoral edema appears to be a more promising ASL perfusion metrics in differentiating high grade glioma from a solitary metastasis.

## INTRODUCTION

Glioblastomas and solitary brain metastases often have similar imaging appearances at conventional MRI. Both lesions show a heterogeneous enhancing mass surrounded by extensive peritumoral edema on T2-weighted images [1-3], leading to wrong classification in more than 40% of cases [4, 5]. As medical staging and treatment decisions of these two types of tumors is vastly different, it is clinically important to distinguish between them with certainty.

In recent years, functional and molecular imaging significantly improve diagnostic specificity and provide an insight into the underlying biological characteristics of brain tumors [6, 7]. Advanced modalities such as spectroscopy imaging [8, 9], diffusion imaging [9-11] and perfusion imaging [11-14] have demonstrated various degrees of success in differentiating glioblastomas from metastases. Perfusion imaging provides non-invasive quantitative methods in assessing tumor vascularity, and can be mainly divided into two techniques: dynamic susceptibility contrast-enhanced perfusion weighted imaging (DSC-PWI) and arterial spin labeling (ASL).

Research has shown that DSC-PWI is limited by susceptibility artefacts and relies on the intravenous injection of contrast agent. However, ASL is a totally non-invasive, perfusion MRI technique that uses magnetically labeled endogenous arterial blood. Studies reveal that ASL perfusion can be utilized as a reliable alternative to dynamic susceptibility contrast perfusion technique [15-18]. Glioblastomas are infiltrative lesions, invading the surrounding white matter [19-21] while metastases are not [22-24]. Therefore, cerebral blood volume (CBV) derived from perfusion imaging can be used by detecting the difference between glioblastoma tumor infiltrative edema and metastatic tumor-free vasogenic edema [20, 22, 25]. However, cerebral blood flow (CBF) deprived from ASL has rarely been studied to differentiate glioblastomas from solitary metastases [26].

Comparing with solitary metastases, we speculated that the CBF values in peritumoral edema of glioblastoma may raise due to angiogenesis of tumor cells. Basing on this, we hypothesized that the difference of CBF value from the edema region close to the enhancing lesion to the area close to the normal appearing white matter might reflect as a gradient — the CBF gradient. Furthermore, we aimed to investigate whether the CBF gradient can be helpful to discriminate glioblastomas from solitary metastases.

## MATERIALS AND METHODS

### Study population

This study continuously enrolled patients with intra-axial brain tumors in the Department of Neurosurgery at our hospital between April 2014 and May 2015. In each patient, MR examinations including conventional MRI and 3D-ASL were performed before surgical operation. All tumor diagnoses had been histologically verified according to the 2007 WHO classification of tumors of the central nervous system. Of the 52 cases, glioblastomas were verified in 24 patients and solitary metastases in 28. Glioblastomas included 22 glioblastoma multiformes and 2 gliosarcomas. Solitary metastates included 18 lung carcinomas, 1 esophageal carcinoma, 2 breast carcinomas, 1 renal carcinoma, 2 colon carcinomas, 1 soft tissue sarcoma, and 3 with unknown origin. Informed consents were obtained from all patients using a protocol approved by local ethics committee in our hospital.

### MR imaging and data processing

All subjects were examined using a 3.0 Tesla MR scanner(Discovery 750, General Electric Medical System, Milwaukee, WI, USA) and an 8 channels high resolution head coil. Foam padding was used to limit the head motion and ear plugs were used to reduce scanner noise. All subjects were told to be relax, and keep eyes open during the examination. Cigarettes, alcohol and acute substance were not allowed before scanning. Conventional MR images included axial T1-weighted images, axial T2-weighted images, axial T2-FLAIR images, axial T2*-weighted images, axial diffusion weighted images, post-contrast axial, coronal, and sagittal T1-weighted images.

Three-dimensional-ASL imaging was performed by pseudo-continuous ASL pulse sequence using a stack of spirals with a background-suppressed 3D fast spin echo imaging sequences [27, 28]. The parameters used were: 512 sampling points on eight spirals, FOV = 24.0 cm, TR/TE = 4653/10.5 ms, post-labeling delay(PLD) =1525 ms, in-plane matrix = 128 × 128, bandwidth = ±62.5KHz, slice thickness = 4.0 mm, inter-slice gap = 0 mm, number of slices = 36, number of averages (NEX) = 3, acquisition time = 4min30s.

3D-ASL data were transferred to an offline workstation (Advantage Workstation, AW4.5; GE Medical Systems). CBF color maps were then postprocessed using GE FuncTool software automatically. CBF was calculated using the following equation:

$$CBF=\frac{\lambda \left(1-e^{\frac{-t_{sat}}{T_{1g}}}\right)}{2\alpha T_{1b}\left(1-e^{\frac{-\tau }{T_{1b}}}\right)}\frac{PW}{PD}e^{\frac{-PLD}{T_{1b}}}$$

where T1b is the T1 of blood (1600ms), T1g is the T1 of gray matter(1200ms), α is the labelling efficiency (0.8), λ is the cortex-blood partition coefficient(0.9), t_sat_ is the time the saturation performed before imaging (2000ms), τ is the labelling duration (1500ms) and PLD is the post-labelling delay time.

One single and experienced neuroradiologist blinded to the final diagnosis analyzed all randomized images. The images were processed equally to measure the CBF values from CBF maps, and T2-weighted and contrast-enhanced T1- images. Peritumoral edema was considered as the area outside the enhancing lesion. It showed hyperintensity on T2 weighted imaging and with no enhancement after intravenous injection on T1 weighted imaging. We selected the most obvious edema sections, than set 3 CBF ROIs in the peritumoral edema from the proximity of the enhancing tumor to the normal-appearing white matter. The ROI next to the enhancing tumor was called G1, the ROI next to the normal-appearing white matter was G3, the ROI of G2 was set in the middle between G1 and G3. We measured the CBF values of all 3 ROIs (CBF _G1_, CBF _G2_, and CBF_G3_), then calculated the CBF gradient as the subtractions CBF_G1_ -CBF_G3_, CBF_G1_ - CBF_G2_, andCBF_G2_ - CBF_G3_.

The maximum CBF value of enhancing lesion(CBFe) and peritumoral edema(CBFp) were also measured. Multiple ROIs(6 to 10) were carefully put in regions with maximum signal on CBF map by visual inspection, and the highest CBF values were selected from above-measured ROIs. This method for the measurement of maximal abnormality provides the highest intraobserver and interobserver reproducibility in CBV measurements [29]. To minimize measurement errors of CBF analysis, uniformly sized ROIs (32 mm^2^) were kept and used for measuring CBF values of the healthy ipsilateral and contralateral normal-appearing white matter. The CBF ratios were calculated by dividing the maximum CBF value in either the enhancing tumor or the peritumoral edema by that of the healthy white matter of the contralateral hemisphere(rCBFe, rCBFp).

### Statistical analyses

The statistical analysis were performed using IBM SPSS Statistics software(version 19.0) and MedCalc software (https://www.medcalc.org/, version 11.4.2.0). The Shapiro-Wilk Test of Normality was used to investigate the distribution of Data. The data were compared between groups with Student's unpaired t-test for continuous variables, Mann-Whitney U test for nonparametric data, and χ2 test for categoric data. The data were presented as mean ± SD, median and range, or frequencies and percentages. A receiver operating characteristic (ROC) curve analysis was used to seek for the best cutoff value permitting discrimination between glioblastomas and metastases. Area under the curve(AUC), Sensitivity, specificity, Youden's Index, positive predict value, and negative predict value were also reported for the optimal thresholds. The level of significant was set at *P* < 0 .05.

## RESULTS

The Clinical information and the CBF values in the glioblastoma and metastasis groups were showed in Table 1. All tumors were supratentorial.

**Figure 1:**
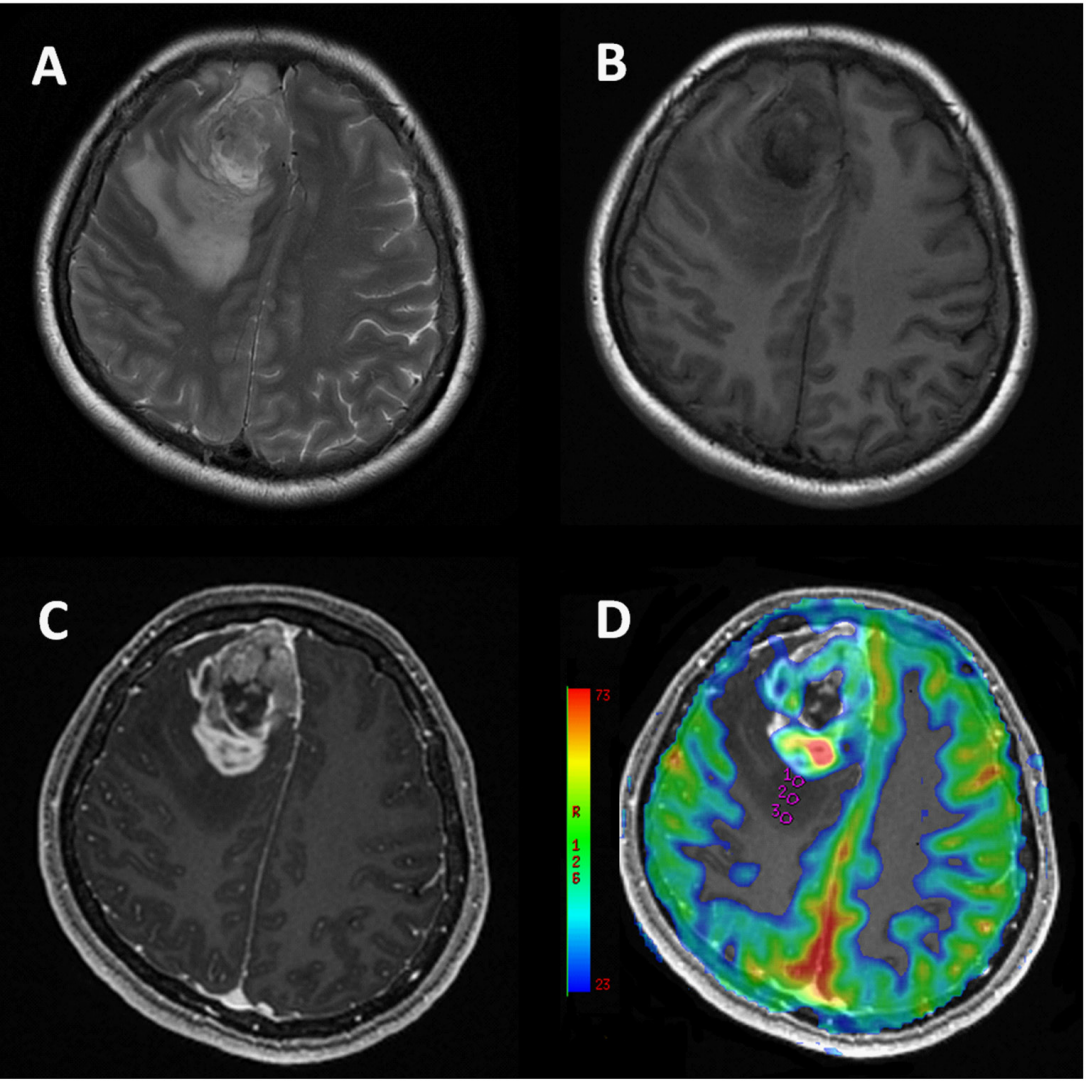
49-year-old female with glioblastoma Conventional T2W **A.**, T1W **B.** and post-contrast T1W **C.** MR images show a heterogeneous enhancing lesion associated with obvious peritumoral edema in right frontal lobe. CBF map **D.** shows ROIs (circles). Measured CBF in ROI1: 14.889 × 10^−3^ ml/100g; ROI2: 10.222 × 10^−3^ ml/100g /s; ROI3: 7.111 × 10^−3^ ml/100g, indicating a CBF gradient in the peritumoral edema from the proximity of the enhancing tumor to the normal-appearing white matter.

**Figure 2:**
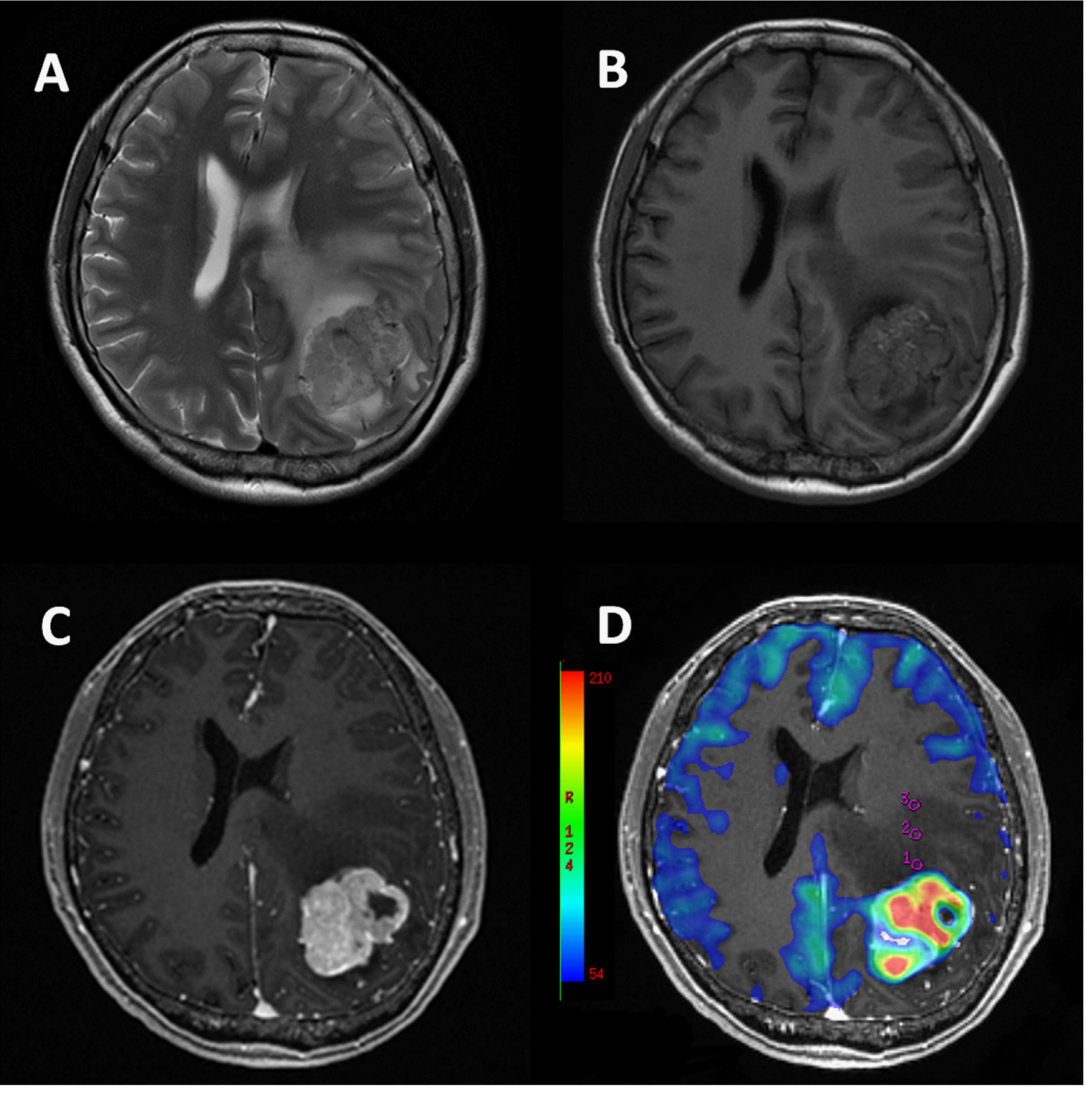
43-year-old female with metastasis from soft tissue sarcoma Conventional T2W **A.**, T1W **B.** and post-contrast T1W **C.** MR images show a heterogeneous mass associated with extensive peritumoral edema in left parietal lobe. CBF map **D.** shows ROIs (circles). Measured CBF in ROI1: 23.111 × 10^−3^ ml/100g; ROI2: 23.778 × 10^−3^ ml/100g /s; ROI3: 24.111 × 10^−3^ ml/100g, suggesting CBF are nearly homogeneous in the peritumoral edema of metastasis.

All CBF gradients(CBF_G1_ -CBF_G2_, CBF_G2_ -CBF_G3_ and CBF_G1_ -CBF_G3_) were significantly higher for glioblastoma than for metastasis (*P* < 0.05) (Table 1 and Figures 1-2). Moreover, glioblastoma also showed significantly higher CBF values in peritumoral edema(CBFp, CBFG1 and CBFp/CBFh) (*P* < 0.05) (Table 1). However, no statistically significant difference was found in CBF values in enhancing lesion and normal appearing white matter (CBFe, CBFn, CBFh and CBFe/CBFh) between groups (*P* > 0.05) (Table 1).

**Table 1:** Clinical information and cerebral blood flow for glioblastoma and Metastases

	Glioblastoma	Metastasis	*P*
***n***	24	28	
**Age**	50.71±13.98	55.06±9.56	0.305
**Sex(% male)**	58.33	53.57	0.730
**Steroid use(%)**	25	26	0.772
**Edema range(mm)**	2.61±0.80	2.56±0.88	0.866
**CBFe**	125.00(73.64∼152.19)	83.65(61.47∼110.96)	0.095
**CBFp**	27.42±10.54	18.05±8.13	0.008
**CBF**_**G1**_	24.21±11.54	15.09±8.13	0.013
**CBF**_**G2**_	18.85±11.44	15.75±6.30	0.336
**CBF**_G3_	17.09±9.03	17.02±5.73	0.982
**CBF**n	23.68±9.34	21.49±9.52	0.520
**CBF**h	25.71±10.20	27.20±12.31	0.718
**CBF**_**G1**_ –**CBF**_**G2**_	3.53±4.75	-0.66±3.41	0.007
**CBF_G2_ –CBF_G3_**	1.76±4.81	-1.28±3.10	0.038
**CBF_G1_ –CBF_G3_**	7.11±5.37	-1.94±2.92	0.000
**CBFe**/**CBFh**	4.79(2.71∼8.52)	3.49(2.35∼8.31)	0.790
**CBFp**/**CBFh**	1.14±0.41	0.70±0.28	0.001

Note—Data are presented as mean ± SD, percentages, n(%), or median(ranges)

CBFe= maximum CBF value in the enhancing lesion, CBFp= maximum CBF value in peritumoral edema, CBFn= mean CBF value in ipsilateral normal-appearing white matter, CBFh= mean CBF value in contralateral healthy white matter, G1= ROI next to enhancing tumor, G2= ROI in middle of peritumoral edema, G3= ROI farthest from enhancing tumor.

**Figure 3:**
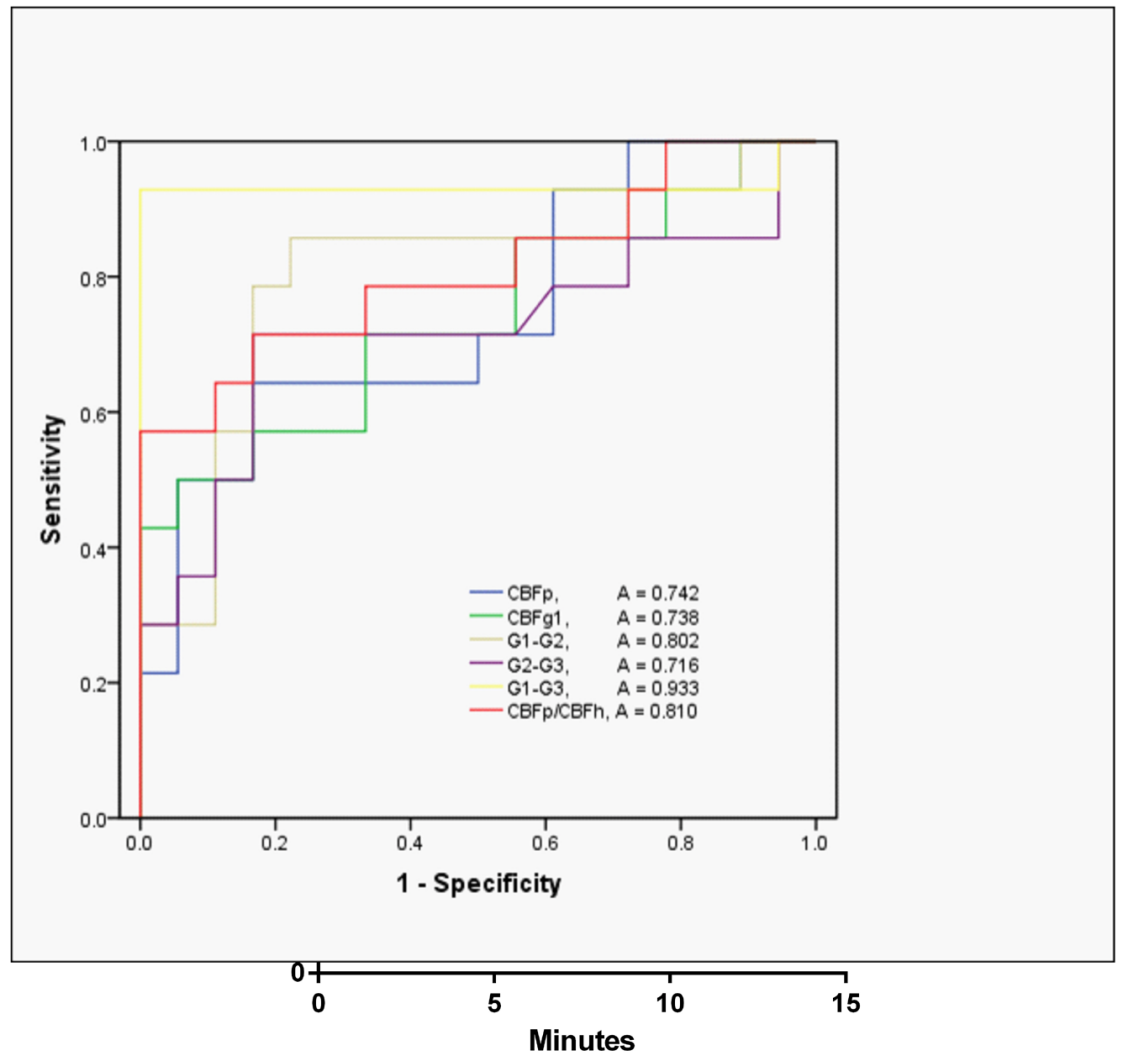
ROC curves for CBF metrics in differentiating glioblastoma from metastases ROC curves: receiver operating characteristic curves; A: area under the curve.

The results of the ROC curve analysis are presented in Table 2 and Figure 3. According to the ROC curve analysis, the AUC of CBF_G1_ -CBF_G3_ was the largest for differentiating glioblastomas from metastases. The optimal cutoff value in differentiation between glioblastomas and metastases was 1.92 (sensitivity, 92.86%; specificity, 100%; positive predictive value, 100%; negative predictive value, 94.7%). In decreasing order of AUC value, the discriminative ability of CBF metrics were CBF_G1_ -CBF_G3_, CBFp/CBFh, CBF_G1_ -CBF_G2_, CBFp(max), CBF_G1_ and CBF_G2_ -CBF_G3_. In the ROC curve analysis, the diagnostic models based on CBF_G1_ -CBF_G3_ had significantly higher AUC than that based on CBF_G1_ and CBF_G2_ -CBF_G3_(*P* = 0.020 and *P* = 0.036, respectively) each alone. However, no significant difference was found from each other in terms of AUC (*P* > 0.052).

**Table 2:** Measures of AUC, cutoff, sensitivity, specificity, Youden's index, PPV and NPV using CBF parameters in the peri-enhancing edema in discrimination of glioblastoma from metastasis with ROC curve analysis

	AUC	Cutoff	Sensitivity	Specificity	Youden	PPV	NPV
**CBFp**	0.742	23.56	64.29	83.33	47.62	75	75
**CBF**_**G1**_	0.738	20.25	50.00	94.44	44.44	87.5	70.8
**CBF**_**G1**_**–CBF**_**G2**_	0.802	1.00	85.71	77.78	63.49	75.0	87.5
**CBF**_**G2**_ **–CBF**_**G3**_	0.716	0.42	71.43	83.33	54.76	76.9	78.9
**CBF**_**G1**_ **–CBF**_**G3**_	0.933	1.92	92.86	100.00	92.86	100	94.7
**CBFp/CBFh**	0.810	1.14	57.14	100.00	57.14	100	75.0

AUC= area under the curve, Cutoff= cutoff value, Youden= Youden's index, PPV= positive predictive value, NPV= negative predictive value.

## DISCUSSION

Differentiating glioblastomas from solitary brain metastases using conventional MRI remains an important unsolved medical problem [30]. Yet, preoperative distinction between these two is of great importance as their therapeutic schedules are totally different. Advanced MRI techniques like perfusion-MRI provides physiologic and hemodynamic information about tumoral vascularity. In this study, we introduced the conception of CBF gradient and investigated the contribution of 3D-ASL perfusion metrics on the discrimination of glioblastomas from solitary metastases.

In glioblastomas, the maximum CBF values in the peritumoral edema were higher than metastases. Moreover, the CBF values in the peritumoral edema of glioblastomas decreased from the proximity of the enhancing tumor to the normal-appearing white matter, while these values showed almost no difference in metastases. Furthermore, the CBF gradient in peritumoral edema served as the best ASL perfusion parameter that enabled distinction between glioblastomas and metastases.

Perfusion-weighted MRI techniques is widely used in quantitative assessment of tumor neoangiogenesis. Hemodynamic perfusion parameters including Cerebral blood volume (CBV) and cerebral blood flow (CBF) have been shown to correlate with tumoral microvascular density as well as expression of vascular endothelial growth factor [31, 32].

In this study, we found both absolute and relative CBFp values were quite effective in differentiating glioblastomas from metastases, while no statistically significant difference was found by using CBFe, in agreement with previous studies [26, 33]. This difference in the peritumoral CBF can be partly explained by the difference in pathophysiology of tumor-related edema. Former researches confirm these results, since the peritumoral edema of metastases represents pure vasogenic edema rich in plasma protein derived from leaking capillaries in or around the metastases [34, 35]. Furthermore, Hossman et.al reported that blood flow measurements in edematous tissue have shown to be decreased due to local compression of the microcirculation by edema [36]. On the contrary, in the peritumoral regions of glioblastomas, the vasculature is relatively impervious as peritumoral edema mix with infiltrative tumor cells.

Moreover, glioblastomas consist of a core tumor lesion surrounded by extensive infiltration of tumor cells that decrease in number from the central to peripheral edema area [20, 21]. Tumor cells tend to invade individually or in small groups between the dense network of neuronal and glial cell processes [20, 37]. The observed CBF gradient in the peritumoral edema of glioblastomas was probably due to this gradient of infiltrate cells; that is, there are more neoplastic cells next to the core lesion than far from the tumor mass [20, 21]. For metastases, the increase in CBF values due to a decline of vasogenic edema from the tumor to the normal white matter was expected. Although results showed no statistically significant, a slight increase was observed from CBF_G1_ to CBF_G3_.

To the best of our knowledge, this is the first study using CBF gradient in the peritumoral edema for differentiating solitary metastases from glioblastomas. According to our result, the CBF gradient is the best CBF parameter that enables distinction between glioblastomas and metastases, with an AUC of 0.933. Similarly, other techniques like diffusion weighted imaging has been used to explore the differentiating characteristics, Pierre et.al reported the ADC gradient in peritumoral edema was of value for differentiating metastases from glioblastomas with a AUC of 0.75 [38]. In contrast, our results show that measurement of the CBF gradient is more accurate than the ADC gradient with relative high sensitivity and specificity(92.86%; 100%). In addition, the use of relative parameters like CBF gradients can ameliorate inter and intra individual differences including spatial effect and steriod effect, which may significantly increase diagnostic efficiency in the study.

In this study, we used ASL, a totally non-invasive perfusion MRI method, instead of contrast perfusion technique. ASL requires no exogenous contrast administration, which makes it more valuable and easy to repeatable. So ASL may be a reliable alternative in patients with renal failure, as they may take risk of gadolinium-associated nephrogenic systemic fibrosis, and in children for whom the intravenous contrast injection may be not easy [17]. Furthermore, 3D pseudocontinuous ASL used in this study combines the advantages of continuous and pulsed ASL approaches, allowing for 3D acquisition, shorter scanning time at 3T and fewer susceptibility artefacts by using a spiral acquisition with fast spin echo techniques [39].

There are several limitations in the current study. First, the sample size was limited to 52 patients and only one blinded observer. Further studies, with larger sample sizes and multiple observers, would be of benefit. Additionally, as multiple factors may influence results of 3D-ASL, such as subject age, blood transit time and choice of PLD time, the fixed PLD used in our study may cause nonrandom bias. This means that individual PLD is needed to validate our present perfusion approach in future researches.

In conclusion, we have investigated CBF values and gradients in peritumoral edema of glioblastoma and a metatasic lesion. Statistically, absolute and relative CBF, and CBF gradient both were useful in differentiating between these two, supporting the hypothesis that ASL perfusion MR imaging can detect infiltration of tumor cells in the peri-enhancing region. Moreover, the CBF gradient in peritumoral edema appears to be a more promising ASL perfusion metrics in differentiating high grade glioma from a solitary cerebral metastasis.
